# Assessing Reptile Conservation Status under Global Climate Change

**DOI:** 10.3390/biology13060436

**Published:** 2024-06-13

**Authors:** Qian Li, Weijie Shao, Ying Jiang, Chengzhi Yan, Wenbo Liao

**Affiliations:** 1 Key Laboratory of Southwest China Wildlife Resources Conservation (Ministry of Education), China West Normal University, Nanchong 637009, China; 2 Key Laboratory of Artificial Propagation and Utilization in Anurans of Nanchong City, China West Normal University, Nanchong 637009, China; 3 College of Panda, China West Normal University, Nanchong 637009, China; 4 School of Ecology and Nature Conservation, Beijing Forestry University, Beijing 100083, China

**Keywords:** climate change, conservation status, distribution ranges, environmental variables, reptiles

## Abstract

**Simple Summary:**

This study uses Maxent models to explore the impact of global climate change on the potential distribution of 5282 reptile species and to estimate their future conservation status. The analysis reveals that over 52.1% of reptile species are experiencing a decrease in their distributional ranges due to global climate change, resulting in a rapid increase in the number of threatened species. However, some reptiles are experiencing an expansion in their potential distribution range under climate change, leading to 91 endangered species being reclassified as least-concern species.

**Abstract:**

Global climate change drives variations in species distribution patterns and affects biodiversity, potentially increasing the risk of species extinction. Investigating the potential distribution range of species under future global climate change is crucial for biodiversity conservation and ecosystem management. In this study, we collected distributional data for 5282 reptile species to assess their conservation status based on distributional ranges using species distribution models. Our predictions indicate that the potential distribution ranges for over half of these species are projected to decrease under different scenarios. Under future scenarios with relatively low carbon emissions, the increase in the number of threatened reptiles is significantly lower, highlighting the importance of human efforts. Surprisingly, we identified some endangered species that are projected to expand their distribution ranges, underscoring the potential positive effects of climate change on some special species. Our findings emphasize the increased extinction risk faced by reptile species due to climate change and highlight the urgent need to mitigate the effects of habitat degradation and human activities on their potential distribution in the future.

## 1. Introduction

Environmental degradation caused by human activity is pushing the planet’s biodiversity toward a sixth mass extinction event [1]. In the last 500 years, over 500 species of terrestrial vertebrates have become extinct or are considered possibly extinct [2,3,4,5]. The extinction risk of species results from a combination of factors, including habitat loss, overexploitation, biological invasions, pollution, toxification, and climate disruption [1,2,3]. In particular, anthropogenic climate change has been widely recognized as a primary threat to biodiversity in the coming century [6,7,8,9,10,11]. Rising temperatures and changes in precipitation patterns may lead to habitat loss and shifting, posing a threat to species survival [12]. Extreme climatic events (e.g., droughts, floods, and high temperatures) may make it difficult for species to adapt, especially for those already adapted to specific climatic conditions. Additionally, climate change may impact vegetation cover and ecosystem structure, altering food chains and species interdependence, and driving species toward endangerment or extinction [13,14,15]. Climate change also affects species’ ranges and migration patterns [16]. Warming temperatures may trigger migration to polar regions or high altitudes, altering native habitats, which presents a formidable challenge for species adapted to specific geographic environments [17]. For species with restricted habitats, climate change may further reduce their adaptive space, exacerbating the risk of endangerment.

There is substantial evidence that the significant effects of anthropogenic climate change on distribution ranges and population extirpations are leading to biodiversity losses worldwide [18,19,20]. The primary impact of climate change on animals is the alteration of their range, resulting in the reduction or loss of suitable habitats, which is the most direct cause of species decline and potential extinction [4,6,7,21]. For example, based on the mean annual dispersal distance to favorable habitats under current conditions and future climate change scenarios, it is predicted that up to 41% of the Australian arid zone gecko (*Gehyra variegata*) population will be unable to colonize favorable microclimates by 2070 [22]. Furthermore, a study on 48 species of lizards in Mexico found that 20% of them will be at risk of extinction by 2080 under climate change, highlighting the urgent need for protective measures [23]. However, climate change affects the distribution patterns of different species in different regions variably; while some species are threatened, others benefit from climate change and continue to expand their ranges [24]. Therefore, assessing the potential impacts of climate change on species distributions is crucial for identifying the most vulnerable species and areas, thereby enabling the prioritization of conservation efforts [25,26,27,28].

Reptiles, characterized by limited dispersal capabilities and as typical ectotherms, are highly vulnerable to climate change because of their temperature-dependent physiological functions [29,30]. Therefore, they serve as an excellent group for studying the effects of climate change. Currently, reptiles are facing significant population declines. Whitfield et al. suggested that climate-driven reductions in the quantity of standing leaf litter, a critical microhabitat for reptiles, were a potential factor contributing to the significant decline in reptile population density in Costa Rican tropical rainforests after 1970 [31]. A growing body of research further confirms that reptiles are at the forefront of global terrestrial vertebrate stress and extinction rates, with over 21% of species listed as threatened [29,30,31,32,33,34]. The distribution and ecological status of reptiles closely reflect rainfall and temperature patterns, and climate change will have a significant impact on their biodiversity, and reptiles adapt to climate change by mainly adjusting their potential ranges [29,30,31,32,33,34]. For instance, numerous reptile species are expanding their potential ranges due to climate change in the Northern Hemisphere [32,33,34]. Nevertheless, over 500 reptile species are projected to become extinct due to a decrease in potential distribution ranges over the current century [30]. Anticipating the potential distribution of reptiles in the face of anthropogenic threats to protect them accordingly is considered a key strategy for biodiversity conservation [30]. Therefore, addressing the impacts of future climate change on the potential distribution ranges of reptile species is crucial to provide recommendations for conservation management.

Due to reptiles’ lack of proactive protection from humans and their limited migration capabilities, we project the distribution pattern of reptiles based on their potential distribution, and estimate their status by predicting the potential distribution ranges for all species under current and future climate scenarios. Our aims are to (1) estimate potential distribution ranges for protecting reptiles under current and future climate scenarios and to investigate the spatial distribution patterns of reptiles globally; and (2) determine the endangered status of all species, analyze changes in endangered status under climate change, and identify priority areas for conservation. We sought to understand how future climatic scenarios could alter the geographic ranges of reptiles and assess the subsequent implications for their extinction risk. This approach allowed us to identify species that may be at increased risk and to propose targeted conservation strategies to mitigate these risks.

## 2. Materials and Methods

Species Distribution Models (SDMs) are invaluable tools for predicting the potential distribution of species in both current and future environmental conditions [35,36]. SDMs have been widely used to predict shifts in the ranges of endangered species under climate change and to inform ecosystem management strategies [37,38,39]. Among the various species distribution models, the MaxEnt model is preferred for its precision in predicted outcomes and its spatiotemporal extrapolation capabilities [40]. The advantages of the MaxEnt model include low sensitivity to collinearity among variables, robustness with limited data points, and the capacity to model intricate relationships among variables [41,42,43]. Consequently, the MaxEnt model has gained widespread application in conservation biology, where it is used to assess habitat suitability, explore species’ ecological niche shifts, and study the effects of global climate change on the geographical distribution of specific species [44,45,46,47]. Given that the number of reptile occurrence records varies from 1 to 1000, it is reasonable to use the MaxEnt model to simulate the potential distribution of reptiles.

### 2.1. Occurrence Records

We conducted a comprehensive search for reptile occurrence records using online databases such as the Global Biodiversity Information Facility (GBIF) “https://www.gbif.org/ (accessed on 1 May 2023)” and data published in primary scientific literature [30]. Initially, we gathered all occurrence records with precise geographic information (i.e., longitude and latitude) from the GBIF for the period 1970–2023. Additionally, we conducted intensive data collection through literature searches and fieldwork to supplement global occurrence records. Notably, fieldwork for our study was not conducted globally. Instead, it was restricted to specific regions of China where data collection was feasible, such as Sichuan, Yunnan, Guizhou, Hunan, Jiangxi, Zhejiang, Anhui, and Hubei Province. Our dataset comprised data for 7679 reptile species, totaling 1,768,550 occurrence records. The distribution of occurrence points was as follows: 40.94% in North America (4760 species), 26.17% in Oceania (4456 species), 3.54% in Africa (4851 species), 0.44% in Antarctica (741 species), 23.65% in South America (4707 species), 17.57% in Europe (1531 species), and 5.64% in Asia (4793 species). This dataset covers 65.5% (7679 out of 11,733) of known reptile species to date, and species without occurrence points are listed in Table S1. However, many rare and small-ranged species were not included due to the scarcity of available occurrence records. We predicted the endangerment status for species with fewer occurrence points based on previous studies, but given that different scenario models were used in this study, we have included the relevant information in the supplementary material (Tables S2–S4). Furthermore, we excluded species with fewer than four records from further analyses, as low sample sizes have been shown to limit the accuracy of species distribution models [48]. Consequently, we simulated potential distribution areas only for species with four or more records, totaling 5282 species in this study.

### 2.2. Environmental Predictors

We obtained a total of 19 bioclimatic variables, including current (1970–2000) and two future (2050s and 2070s) scenarios, from the WorldClim database (https://www.worldclim.org/, accessed on 1 August 2023) at a spatial resolution of 10 min (approximately 18.5 km at the equator). For future scenarios, bioclimatic data for the 2050s and 2070s represent the mean values from 2041 to 2060 and 2061 to 2080, respectively. To project future climate change, we used predictions from general circulation models (GCMs) based on Shared Socioeconomic Pathways (SSPs) scenarios, introduced as part of the Coupled Model Intercomparison Project Phase 6 (CMIP6) by the IPCC [49]. Specifically, we utilized the BCC-CSM2-MR climate system model developed by the Beijing Climate Center [50]. We selected two scenarios for this study: SSP2-4.5 and SSP5-8.5. SSP2-4.5 represents a scenario with slower population growth stabilizing by mid-century, moderate economic growth, balanced energy use, moderate technological innovation, and moderate reductions in future GHG emissions due to some, but not strong, climate policies [51]. In contrast, SSP5-8.5 describes a scenario with rapid population growth until the end of the century, rapid economic growth, high technological innovation, increased energy use, continued rise in GHG emissions, and a lack of strong climate policy [51]. By using these two scenarios, we aimed to capture a spectrum of potential future conditions, from moderate to severe climate impacts.

To address multicollinearity among climatic variables, we conducted a Pearson correlation analysis and excluded variables with correlations exceeding 0.8 (Pearson r > 0.8). Subsequently, we calculated the variance inflation factor (VIF) for the remaining variables using the ‘vif’ function in the ‘usdm’ R package and excluded those with VIF values exceeding 10 in R Version 4.0.1 [52,53]. Ultimately, 10 climate variables were retained for further analysis. These variables were mean diurnal range (BIO2), temperature seasonality (BIO4), max temperature of warmest month (BIO5), mean temperature of wettest quarter (BIO8), mean temperature of driest quarter (BIO9), annual precipitation (BIO12), precipitation of driest month (BIO14), precipitation seasonality (BIO15), precipitation of warmest quarter (BIO18), and precipitation of coldest quarter (BIO19). Data on elevation were derived from Earthdata (https://www.earthdata.nasa.gov/, accessed on 1 August 2023).

### 2.3. Species Distribution Models

All modeling was conducted using MAXENT Version 3.4.3, which was chosen for its ability to rely on presence points exclusively and manage limited occurrence data for some species [48,53,54]. For each species, 75% of occurrence points were randomly selected as the training dataset for model construction, while the remaining 25% served as the validation dataset. We evaluated weights using the Jackknife method and generated receiver operating characteristic (ROC) curves with a repetition number of 10. The maximum number of background points was set to 100,000, and we selected the Logistic output type. The Area Under the Curve (AUC) of ROC curves was employed to assess the model’s predictive accuracy [55]. Utilizing ArcGIS 10.8’s transformation tool, we converted the average results of the seven model predictions from ASCII-encoded files to a raster format [48]. These results were reclassified using Jenks’ natural breaks and categorized into four classes based on distribution values and ranges: non-habitability zones, low-habitability zones, medium-habitability zones, and high-habitability zones. The sum of low, medium, and high fitness zones was defined as the potential distribution range [56,57].

### 2.4. Endangerment Assessment

Changes in species richness are primarily driven by shifts in species ranges. To address the distribution pattern and range change of reptiles, we mapped the species richness of reptiles for each 1° cell under current and future scenarios. We then calculated changes in species richness in each cell by comparing species richness under future scenarios with species richness at the current stage, aiming to determine the effects of climate change on the distribution patterns of reptiles.

In this study, changes in IUCN endangered species rankings were determined by the rate of change in the range of reptile ranges. The assessment of potential future endangerment for reptiles followed the methodology outlined in the IUCN Endangered Species Criteria A3(C) [58,59,60]. The assessment was based on the following criteria: (1) a species is classified as extinct (EX) when it is on the brink of losing 100% of its potential range; (2) a species is categorized as Critically Endangered (CR) when it is on the verge of losing 80% of its potential range; (3) species are designated as Endangered (EN) when they are on the brink of losing 50% of their potential range; (4) species are classified as Vulnerable (VU) when they are on the brink of losing 30% of their potential range; (5) species are considered Near Threatened (NT) when they are on the verge of losing 10% of their potential range; and (6) the remaining species are categorized as Least Concern (LC).

Given that the modeled sample data are derived from occurrence data, species with limited occurrence points may indicate difficulty in collection or a small population size in the wild. Considering the overall declining trend of global biodiversity under climate change pressure, these species may face more serious threats. In our study, for species not included in the simulation of potential distribution areas, we applied the following treatment: For reptiles with three occurrence points, we calculated the area enclosed by these points. If the area was less than 100 square kilometers, the endangerment status was elevated by three levels, approaching extinction; if the area was less than 500 square kilometers, the status rose by two levels; if the area was less than 10,000 square kilometers, the status increased by one level (Table S2). For reptiles with two records, we computed the distance between the two points. When the distance was less than 10 km, the endangerment rating increased by three levels; when it was less than 50 km, the rating increased by two levels; and when it was less than 500 km, the rating increased by one level (Table S3). For species with only one record, their risk increased by three levels (Table S4). For these species with limited sample sites, we assumed that their endangered status remained unchanged from the 2050s to 2070s.

## 3. Results

**Figure 1:**
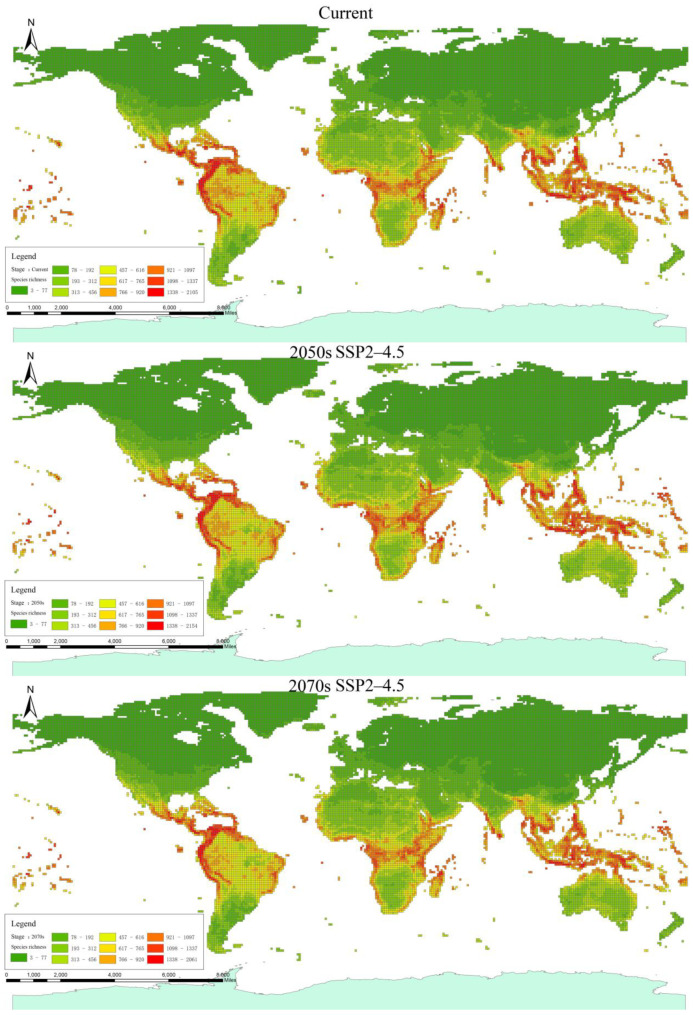
Spatial pattern for species richness changes under future climate scenario SSP2–4.5.

The MaxEnt model exhibited excellent predictive performance for all analyzed species (AUC train = 0.99, AUC test = 0.98, 2050s; AUC train = 0.99, AUC test = 0.99, 2070s). Therefore, the MaxEnt model maintains high accuracy and reliability in predicting the distribution range of reptiles under global climate change. Regarding reptile species richness (Figure 1 and Figure S1), there is a decreasing trend under global climate change. Based on the projected range change of species, South America, North America, the central and eastern coast of Africa, Southeast Asia, and Australia are expected to experience the highest amount of habitat loss, with China and other Asian regions being the least affected (Table 1).

**Figure 2:**
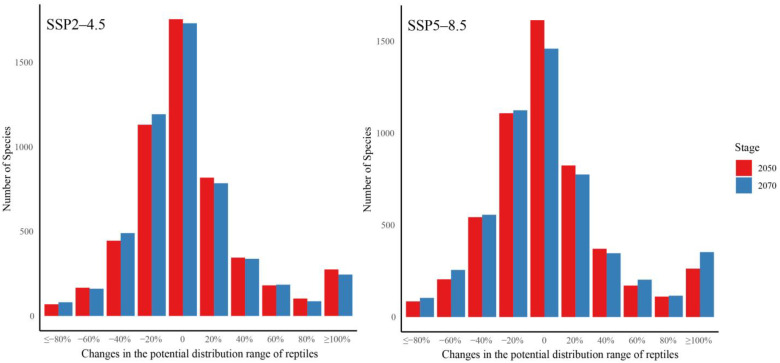
The percentage change in climatically suitable habitats for IUCN red list species in 2050 and 2070 based on MaxEnt. Negative numbers on the x-axis indicate a loss in suitable habitats.

Projecting the current MaxEnt model of species onto climate change scenarios indicates that over 52.1% of reptile species will likely experience a contraction of potential distribution ranges from the present time to 2050 and 2070 under SSP2-4.5 and SSP2-8.5 scenarios (Figure 2). We found that 52.08% (2050s, SSP2-4.5, Table S5) to 53.61% (2070s, SSP2-4.5, Table S6) of the species are projected to have reduced potential ranges, while 52.72% (2050s, SSP5-8.5, Table S5) to 55.0% (2070s, SSP5-8.5, Table S6) are projected to have reduced ranges. Over 80% of the potential distribution ranges of 32 and 38 reptile species were projected to decrease from the 2050s and 2070s under the SSP2-4.5 scenario and were regarded as critically endangered species. Meanwhile, the potential distributions ranging from 50% to 80% of 204 species (2050s, SSP2-4.5) and 200 species (2070s, SSP2-4.5) were projected to decrease and were regarded as endangered species.

**Figure 3:**
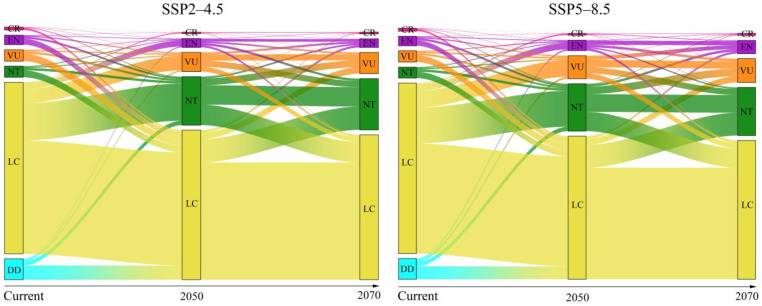
Changes in the IUCN Red List categories for reptiles over time. The x-axis represents the temporal scale, where current refers to the current species conservation status according to the IUCN, while 2050 and 2070 are the predicted conservation statuses according to the change in distribution range. The thickness of the line represents the number of species. Extinct (EX); Critically Endangered (CR); Endangered (EN); Vulnerable (VU); Near Threatened (NT); Least Concern (LC); Data Deficient (DD).

Additionally, our results revealed that 445 species and 489 species will lose ranging from 30% to 50% of their current suitable habitats from the current time to the 2050s and 2070s under the SSP2-4.5 scenario and become vulnerable species. There are 1126 species (2050s) and 1190 species (2070s) under the SSP2-4.5 scenario whose potential distribution ranges were ranging from 10% to 30%, which became near-threatened species. Finally, the potential distribution ranges of 944 species (2050s) and 915 species (2070s) were projected to decrease less than 10%, as well as the potential distribution ranges of 2531 species (2050s, SSP2-4.5) and 2450 species (2070s, SSP2-4.5) expanding, which were regarded as least-concern species. We also predicted similar results under the SSP5-8.5 scenario. In total, the number of all reptile species for threat was projected to increase by 22.70% in the 2050s and 30.99% in the 2070s under the SSP2-4.5 scenario, while it was projected to increase by 49.73% in the 2050s and 64.68% in the 2070s under the SSP5-8.5 scenario (Table S7). In particular, the number of near-threatened species was projected to increase by 336.43% (2050s, SSP2-4.5) and by 368.37% (2070s, SSP2-4.5) (Figure 3).

Our findings indicate that reptiles will confront heightened threats to their survival across different socioeconomic pathways in the future. Particularly notable is the substantial increase in the number of near-threatened (NT) species, surpassing three times the current count across all socioeconomic pathway scenarios. Consistently, our simulations demonstrate an elevated risk of extinction for many reptile species due to climate change, potentially driving some species towards extinction. Furthermore, we observed significantly lower population growth among stressed reptiles under the SSP2-4.5 scenario, suggesting that mitigation efforts, such as reducing greenhouse gas emissions, implementing nitrogen fixation strategies, and curtailing deforestation, could notably decelerate the rate of species extinction.

Meanwhile, we also predicted an expansion of the potential distribution ranges of reptile species, revealing that many species show strong adaptation to future climate change. We found that the potential distribution ranges of 17 and 25 critically endangered species of reptiles were projected to increase in the 2050s under SSP2-4.5 and SSP5-8.5 scenarios, respectively, which were classified as least-concern species. Meanwhile, the potential distribution ranges of 73 and 66 endangered species of reptiles were projected to increase in the 2070s under SSP2-4.5 and SSP5-8.5 scenarios, respectively, and were also classified as least-concern species.

## 4. Discussion

Our study on the potential distribution of 5282 reptile species offers valuable insights into current reptile conservation efforts and the challenges posed by future climate change. We observed significant shifts in the global distribution patterns of reptiles due to climate change, with over 50% of reptile potential ranges contracting under both the SSP2-4.5 and SSP5-8.5 scenarios. Moreover, the number of stressed species has surged, with over three times as many species classified as Least Concern (LC) at present. Concurrently, certain reptile species have benefited from climate change, experiencing expanded potential ranges. Notably, approximately 20 Critically Endangered (CR) species and nearly 70 Endangered (EN) species have transitioned to Least-Concern (LC) status. Furthermore, we observed a notable reduction in the population growth of stressed reptiles under scenarios with lower carbon emissions. These findings underscore the pivotal role of potential distribution, influenced by habitat degradation and human activities, in shaping reptile biodiversity under the pressures of global climate change.

Global climate change is widely recognized as a significant driver of changes in wildlife species’ morphology, habitat use, distribution ranges, and conservation status [2,3,4,61,62,63,64,65,66]. Temperature and precipitation patterns are altered by climate change, leading to shifts in wildlife’s original natural environments and prompting their migration to areas with more favorable climatic conditions for survival [59,65]. Climate change not only directly impacts wildlife but also induces changes in other ecological factors associated with the environment, indirectly influencing species distribution [67]. For instance, changes in vegetation patterns, which not only serve as a wildlife habitat but also as a food source, can alter wildlife distribution areas [16,33]. Species respond to climate change through behaviors such as migration and dispersal, and the extent of the impact on species distribution depends on their dispersal abilities. Species with high dispersal abilities are more adaptable, and their distribution ranges may expand with the shifting of their boundaries when climate change falls within their tolerance levels [24]. While climate warming can expand the distribution boundaries of many species, providing increased space for survival and reproduction, it may also introduce potential negative impacts, such as challenges in timely adaptation to environmental changes within new ranges and adjustment to new interspecific competition [68]. In summary, climate change affects species distributions, and determining the overall advantages and disadvantages of such changes for species remains complex and context-dependent.

Studying animals’ habitat use under climate change is crucial because it is closely linked to their distribution ranges [4]. Previous research has highlighted the impact of climate change on various animal groups, resulting in significant contraction of distribution ranges. For instance, more than 79% of the distribution range of mountain vipers has shown substantial contraction, potentially leading to extinction in the eastern Mediterranean [69]. Similar trends of distribution range reduction have been observed in amphibians in the Near and Middle East due to projected climate warming [70,71]. Habitat suitability for alpine birds is forecasted to decline by 36.83% to 60.10%, accompanied by an upward range shift [72]. However, a prior study has indicated that habitat use by reptiles within protected areas is expected to increase, while habitats outside protected areas may be lost under climate change [30]. In our study, we observed that habitat ranges for most reptile species are anticipated to decrease under climate change, resulting in diminished distribution ranges. It is imperative to address the impacts of human activities on endangered species, as these species facing climate change are likely to experience reductions in their distribution ranges, potentially leading to extinction [73,74]. Moreover, habitat fragmentation may impede the expansion of species’ distribution ranges by hindering dispersal among young individuals [75,76,77]. Therefore, it is essential to establish effective protected areas for endangered reptile species under climate change.

Reptiles, as poikilothermic animals, have their body temperature influenced by environmental conditions, which in turn impacts their potential distribution ranges and survival [78,79]. Variations in environmental temperature can restrict the potential distribution of reptiles, particularly due to declining population density in excessively high temperatures [80]. Moreover, the escalation in greenhouse gas emissions contributes to global climate warming [81]. Consequently, future climate change is expected to diminish the distribution range of reptiles, driven by rising temperatures leading to reduced suitable habitat areas [82,83]. Our research unveils a notable decrease in potential distribution areas for the 2050s and 2070s, signaling that heightened temperatures under climate change could precipitate a decline in reptile distribution in the future. Nevertheless, it is plausible that we may underestimate species’ range expansions in response to climate warming if we project distribution without factoring in dispersal capacity.

Under global climate change, reptile distribution ranges and conservation statuses exhibit a notable decline. Our findings reveal a significant ongoing contraction in reptile distribution ranges from the present to the 2050s and 2070s driven by temperature increases that impact environmental suitability for most species. To our knowledge, our study provides a comprehensive assessment of the impact of climate change on reptile distribution ranges and conservation statuses. Previous research has similarly highlighted anticipated changes in distribution ranges and conservation statuses under future global climate warming, attributing them to decreased distribution ranges and an augmented number of endangered species [6,7,18,30]. For example, despite future protected areas in Morocco, reptile distribution ranges may still decrease as the region remains highly susceptible to global climate change [84,85]. In our investigation, we estimate that 52.1% of reptile species face a heightened extinction risk under climate change throughout the ongoing century. Consequently, we advocate for robust conservation strategies that prioritize ecologically representative and well-connected protected areas. Additionally, we recommend reducing human activities in the conservation habitats of endangered species, as these species are particularly vulnerable to high extinction risks.

An important aspect of our findings is the distinction between changes in species number (quality) and changes in potential distribution range (quantity). Our results indicate that, while many reptile species experience significant shifts in potential distribution ranges under future climate scenarios, the overall number of species shows comparatively less change. This suggests that climate change is more likely to affect the locations where species can live rather than the total number of species that can survive. The contraction or expansion of potential ranges implies that species may need to relocate to maintain viable populations, highlighting the importance of habitat connectivity and the establishment of migration corridors [15,18,84]. Consequently, conservation efforts should focus not only on preserving current habitats but also on facilitating the movement of species to new, more suitable areas as their current habitats become less viable due to climate change.

However, our assessment of endangerment ratings heavily relies on changes in potential distributional ranges, which has inherent limitations. Failing to consider the combined effects of factors such as biological invasions, anthropogenic disturbances, population structure, and inter-organism interrelationships may have contributed to our relatively conservative assessment of reptile extinction risk. Future studies could expand the analyses to incorporate these factors, thereby enhancing a more comprehensive and accurate understanding of endangerment statuses.

## 5. Conclusions

We investigate the impact of global climate change on the potential distribution of reptile species and observe a considerable reduction in their potential distribution area for the 2050s and 2070s. Our findings add to the growing body of evidence suggesting that future global climate change poses an escalating threat to reptiles. Specifically, we emphasize the role of climate warming in heightening the extinction risk of reptile populations. Future research endeavors could further explore the specific mechanisms through which changing climate factors, such as temperature and precipitation, influence reptile ecology and behavior, thereby enhancing our understanding of these dynamics.

## Figures and Tables

**Table 1:** Species richness of each continent under current and future climate scenarios.

Continent	Current	SSP2-4.5		SSP5-8.5	
		2050s	2070s	2050s	2070s
North America	4765	4754	4775	4751	4743
South America	4711	4671	4694	4700	4711
Antarctica	743	792	785	768	749
Asia	4798	4762	4812	4766	4767
European	1565	1597	1563	1506	1553
Oceania	4461	4385	4384	4397	4356
Africa	4856	4828	4840	4863	4833

## Data Availability

The data presented in this study are available on request from the corresponding author.

## Supplementary Materials

The following supporting information can be downloaded at https://www.mdpi.com/article/10.3390/biology13060436/s1: Figure S1: Spatial pattern for species richness changes under future climate scenario SSP5-8.5; Table S1: Species without occurrence points; Table S2: Species with three occurrence points; Table S3: Species with two occurrence points; Table S4: Species with one occurrence point; Table S5: List of species range area reductions in the 2050s; Table S6: List of species range area reductions in the 2070s; Table S7: List of species range area expansions.
