# Supplementary material for: Assessing Reptile Conservation Status under Global Climate Change
Source: Biology (Basel). 2024 Jun 13;13(6):436. doi: 10.3390/biology13060436 (PMC11200438; doi:10.3390/biology13060436)
Supplement: Supplementary file 1 [file biology-13-00436-s001.zip › Supplementary Material.pdf]

Supplementary Material

Assessing Reptile Conservation Status under Global Climate Change

Qian Li [1,2,3], Weijie Shao [1,2,3], Ying Jiang [1,3,4], Chengzhi Yan [1,2,3] and Wenbo Liao [1,2,3,*]

[1] Key Laboratory of Southwest China Wildlife Resources Conservation (Ministry of Education), China West Normal University, Nanchong 637009, China
[2] Key Laboratory of Artificial Propagation and Utilization in Anurans of Nanchong City, China West Normal University, Nanchong 637009, China
[3] College of Panda, China West Normal University, Nanchong 637009, China
[4] School of Ecology and Nature Conservation, Beijing Forestry University, Beijing 100083, China
* Correspondence: liaobo_0_0@126.com

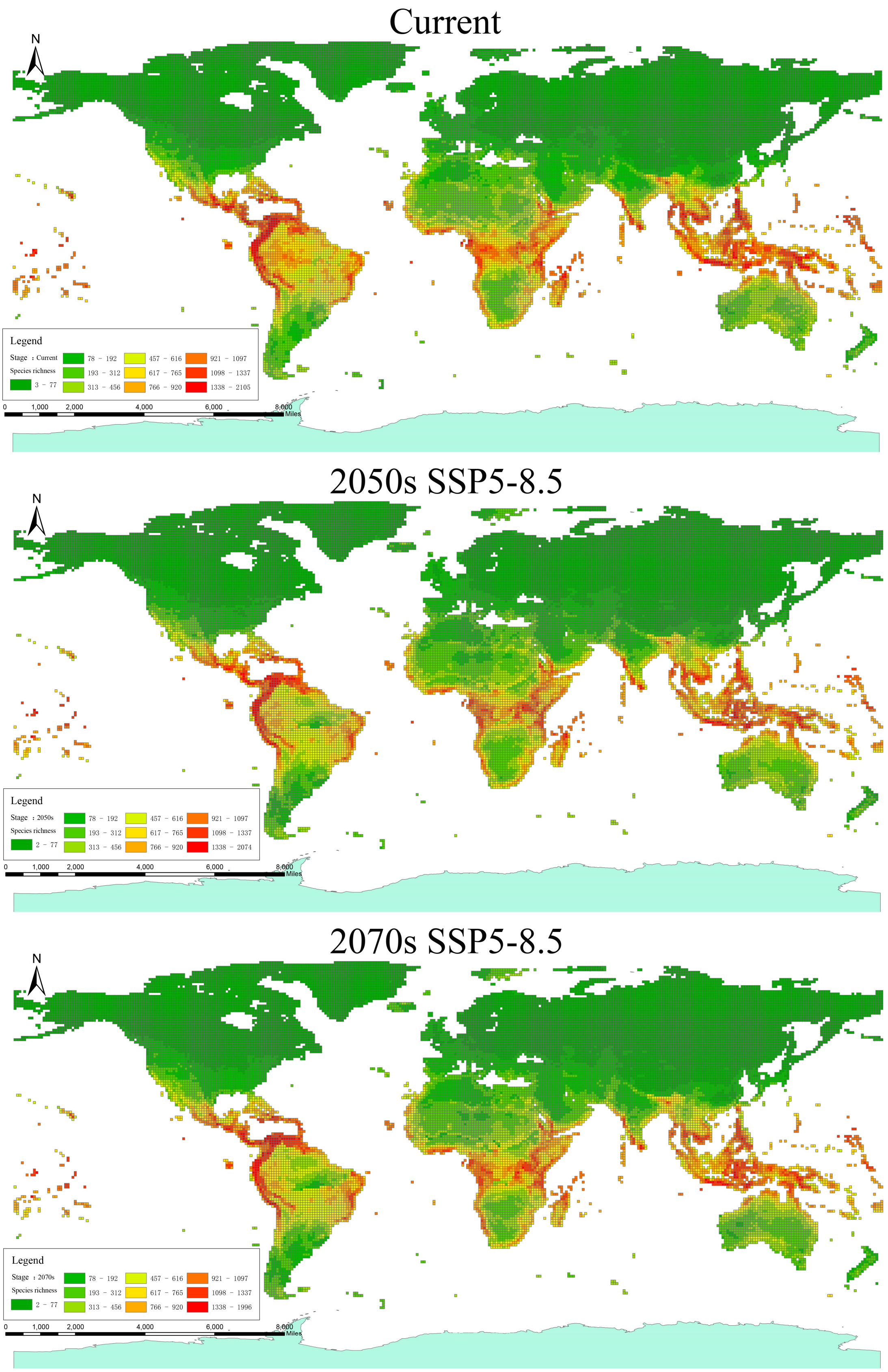

Figure S1: the distribution pattern of Reptiles under current and future scenarios.
